# Impact of Diagnosed Mental Health Disorders on Postoperative Outcomes After Achilles Tendon Repair

**DOI:** 10.1177/24730114261457093

**Published:** 2026-06-29

**Authors:** Ahad A. Kesaria, Saadi M. Irfan, Tristan X. Nguyen, Jeffrey L. Shi, Verdinand C.B. Ruelos, William M. Weiss

**Affiliations:** 1 John Sealy School of Medicine, The University of Texas Medical Branch, Galveston, TX, USA; 2 Department of Orthopaedic Surgery and Rehabilitation, The University of Texas Medical Branch, Galveston, TX, USA

**Keywords:** Achilles tendon, Achilles tendon repair, mental health, postoperative outcomes, postoperative complications

## Abstract

**Background:**

This study evaluated the association between diagnosed mental health disorders and postoperative outcomes following Achilles repair.

**Methods:**

A retrospective cohort study was performed using the TriNetX database over a 20-year period. Patients were divided into 2 cohorts based on whether they had a documented mental health disorder (eg, anxiety or depression) 1 year preceding surgery. Cohorts were propensity score matched by demographics such as age, gender, body mass index, diabetes, and hypertension. Outcomes were measured 90 days, 3 years, and up to 10 years using odds ratios with 95% CIs. χ^2^ analysis was performed with significance noted at *P* <.05.

**Results:**

Matched cohorts had a mean age of 48.5 years in the mental health cohort and 49.6 years in the control cohort, with 47.4% vs 47.1% female patients, respectively. A total of 1841 patients were included in each cohort. Within 90 days postoperative, patients with diagnosed mental health disorders demonstrated several significant outcomes such as higher odds of orthopaedic aftercare (*P* = .032), infection following a procedure (*P* < .0001), postoperative pain (*P* < .0001), emergency department visit (*P* < .0001), ankle pain (*P* = .004), cellulitis of lower limb (*P* < .0001), and foot pain (*P* = .009). Within 3 years postoperative, these patients had significantly higher odds of knee pain (*P* = .008), ankle osteoarthritis (*P* = .003), Achilles tendonitis (*P* = .008), and abnormalities of gait and mobility (*P* = .020). Within 10 years postoperative, these patients had significantly higher odds of ankle osteoarthritis (*P* = .006).

**Conclusion:**

Diagnosed mental health disorders are associated with increased short- and long-term adverse postoperative outcomes in patients who underwent Achilles repair at a population level. These associations included increased infection rates and pain, as well as later musculoskeletal diagnoses such as ankle osteoarthritis and abnormalities with gait.

**Level of evidence:**

Level III, retrospective, comparative cohort study.

## Graphical abstract

**Visual Abstract:**
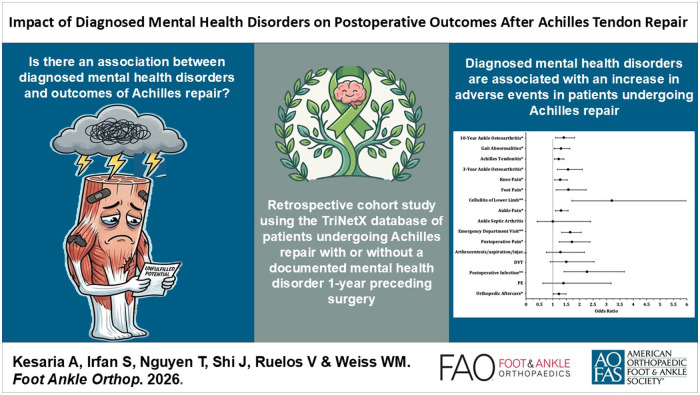
This is a visual representation of the abstract.

## Introduction

Achilles tendon rupture (ATR) is one of the most common tendon injuries in adults, accounting for approximately 10% of all tendon and ligament injuries and often leading to substantial functional impairment.^1^ Recent epidemiologic data demonstrate that the incidence of ATR continues to rise, with estimates approaching 40 per 100 000 person-years.^1^ The injury often results in significant weakness, impaired plantarflexion strength, and limitations in returning to prior activity levels.^2^ For active individuals, surgical repair is frequently pursued to restore tendon continuity and optimize long-term functional recovery.^2^

Despite being a commonly performed procedure, Achilles tendon repair carries meaningful postoperative risks. Reported complications include surgical site infection, wound dehiscence, sural nerve injury, and re-rupture, each of which can substantially hinder recovery and delay return to function.^3,4^ Although advances in surgical technique and rehabilitation protocols have improved outcomes, these complications remain clinically significant. Identifying patient-specific factors that predispose individuals to adverse outcomes is therefore critical to optimizing perioperative management and long-term recovery.^5^

Mental health disorders such as depression and anxiety are highly prevalent in orthopaedic populations and often exceed rates observed in the general public.^6^ Although approximately 5% of adults experience major depressive disorder and 3% to 4% experience generalized anxiety at a given time, orthopaedic patients show substantially higher rates of psychological comorbidity.^7^ Studies in trauma, elective orthopaedic surgery, and foot and ankle cohorts report that 30% to 45% of patients have a diagnosable psychiatric condition, with depression and anxiety being the most common.^7-9^ These data highlight that a considerable proportion of patients undergoing musculoskeletal procedures enter surgery with underlying mental health conditions that may influence their postoperative course. We hypothesized that patients with diagnosed mental health disorders would demonstrate increased odds of postoperative complications and health care utilization following Achilles tendon repair compared with patients without diagnosed mental health disorders.

Growing evidence suggests that mental health disorders can adversely influence surgical outcomes.^8^ Depression and anxiety have been linked to increased morbidity, slower rehabilitation, and poorer functional recovery across various orthopaedic procedures.^10^ However, most prior investigations have focused primarily on patient-reported outcomes—such as pain, satisfaction, or subjective functional measures—rather than objective postoperative endpoints. Structural and biomechanical sequelae, including infection, impaired tendon healing, long-term joint degeneration, and gait abnormalities, have been examined far less frequently. Consequently, the extent to which preoperative mental health diagnoses influence measurable postoperative outcomes following Achilles tendon repair remains unclear.

Given the substantial prevalence of psychological comorbidities in orthopaedic patients and the recognized influence of mental health on recovery, understanding whether these conditions affect outcomes after Achilles repair is clinically important. Despite well-documented associations between mental health disorders and postoperative morbidity in other orthopaedic procedures, large-scale analyses on their impact on objective, short- and long-term complications following Achilles tendon repair remain limited. To address this gap, the present study uses a large, multiinstitutional database to compare postoperative outcomes in patients with and without diagnosed mental health disorders undergoing Achilles tendon repair. Our goal is to provide a comprehensive, longitudinal assessment of mental health as an associated factor for both early and delayed adverse outcomes in this population.

## Methods

Deidentified patient information was obtained through TriNetX, a global federated electronic health record research network that aggregates patient data from participating health care organizations (HCOs) and enables large-scale retrospective cohort analyses. These HCOs consist of hospitals and provider groups that share data including diagnoses, procedures, medications, laboratory values, and genomic profiles. Because all data within TriNetX are deidentified in compliance with federal and institutional policies, the use of this database is exempt from institutional review board (IRB) approval and patient consent. For this study, data were drawn from the Research network, which included 94 HCOs, each contributing records. The data set covered encounters between September 21, 2004, and September 21, 2024.

Two cohorts were established for this study. Both groups included patients who underwent primary open or percutaneous Achilles tendon repair, identified by *CPT* (*Current Procedural Terminology*) codes 27650 and 1014587. Cohort A consisted of patients with a documented mental health diagnosis within 1 year preceding surgery, as defined by *International Classification of Diseases, Tenth Revision* (*ICD-10*) coding. Eligible diagnoses included major depressive disorder (single or recurrent episodes, with or without psychotic features), dysthymia, bipolar disorder (current or partial remission episodes, mild to severe), adjustment disorders with anxiety or depressed mood, generalized anxiety disorder, phobic anxiety disorders, and other specified or unspecified anxiety disorders. Cohort B comprised patients who underwent Achilles tendon repair without any of the mental health diagnoses. To reduce confounding, propensity score matching was used to balance demographic and medical factors.

Following 1:1 propensity score matching, outcomes between cohorts were compared using χ^2^ testing within the TriNetX platform. Odds ratios (ORs) with 95% CIs were generated, and standard mean differences were reported to assess covariate balance. Matching was performed on demographic variables including age at index surgery, sex (male, female), race (White, Black or African American, Asian, American Indian or Alaska Native, Native Hawaiian or other Pacific Islander, other, or unknown), and ethnicity (Hispanic or Latino, not Hispanic or Latino, or unknown). Medical comorbidities incorporated in the matching process included hypertension, type 2 diabetes mellitus, nicotine dependence, alcohol-related disorders, opioid-related disorders, obesity, antidepressant use, and osteoarthritis (Supplementary Table 1).

Follow-up intervals of 90 days, 3 years, and up to 10 years were selected to capture short-term postoperative complications, intermediate-term musculoskeletal diagnoses, and longer-term associations. The 10-year interval represents a maximum outcome assessment window rather than continuous follow-up for all patients. Short-term outcomes evaluated within 90 days following Achilles tendon repair included orthopaedic aftercare, infection following a procedure, postoperative pain, emergency department visits, ankle pain, cellulitis of the lower limb, foot pain, pulmonary embolism, deep vein thrombosis, arthrocentesis/aspiration/injection, and ankle septic arthritis. Orthopaedic aftercare was defined using *ICD* coding corresponding to postoperative follow-up, rehabilitation, or aftercare encounters related to musculoskeletal treatment. Long-term outcomes were assessed at both 3 and 10 years postoperatively. Outcomes analyzed through 3 years included knee pain, ankle osteoarthritis, Achilles tendonitis, gait abnormalities, ACL rupture, repeat Achilles tendon repair, and secondary Achilles tendon repair. At 10 years, outcomes included ankle osteoarthritis, short Achilles tendon, and calcific tendonitis of the Achilles (Supplementary Table 2). Statistical significance was set at *P* <.05. Although laterality can be specified for certain *CPT* and diagnosis codes within the TriNetX platform, restricting outcomes to the ipsilateral operative limb substantially reduced cohort size and statistical power. Therefore, postoperative outcomes were analyzed at the patient level rather than limited to the operative extremity. All outcomes were evaluated in an exploratory and hypothesis-generating manner, and no formal adjustment for multiple comparisons was performed. Laterality completeness was assessed by determining the proportion of index procedures linked to laterality-specific *ICD-10* diagnosis codes. Follow-up within the TriNetX network was evaluated using the platform’s follow-up metrics, which quantify the duration of observed patient activity within the network rather than requiring continuous enrollment.

## Results

A total of 18 056 total patients met the criteria for inclusion using the TriNetX database query, with 2151 of those patients having a documented mental health disorder who underwent primary Achilles tendon repair and 15 905 patients without a recorded mental health diagnosis who underwent the same procedure. After performing a 1:1 propensity score matching within the TriNetX software, 1841 patients remained in each cohort for analysis.

Following matching, demographic and clinical variables were well balanced between groups, confirmed by χ^2^ testing and standardized mean difference (SMD) values. Patients who underwent Achilles tendon repair with diagnosed mental health disorders (cohort A) had a mean age at index of 48.5 ± 14.0 years, compared with 49.6 ± 14.3 years in patients without a mental health diagnosis (cohort B). Both cohorts had similar sex distributions, with females representing 47.4% of cohort A and 47.1% of cohort B, and males comprising 44.5% and 43.5%, respectively (*P* > .05 for both). After matching, the White race remained the most prevalent in both cohorts, representing 70.1% of cohort A and 71.4% of cohort B. All demographic and clinical variables demonstrated standardized mean differences <0.1, confirming appropriate balance following matching (Supplementary Table 1). Laterality-specific *ICD-10* diagnosis codes were available for 74.8% of index procedures, whereas 25.2% lacked laterality designation. Within the propensity-matched cohort used for the 10-year outcome analysis, the mean follow-up duration within the TriNetX network was 1442.6 days (3.95 years) in the mental health cohort and 1391.4 days (3.81 years) in the control cohort.

### Postoperative Outcomes

**Figure 1.**
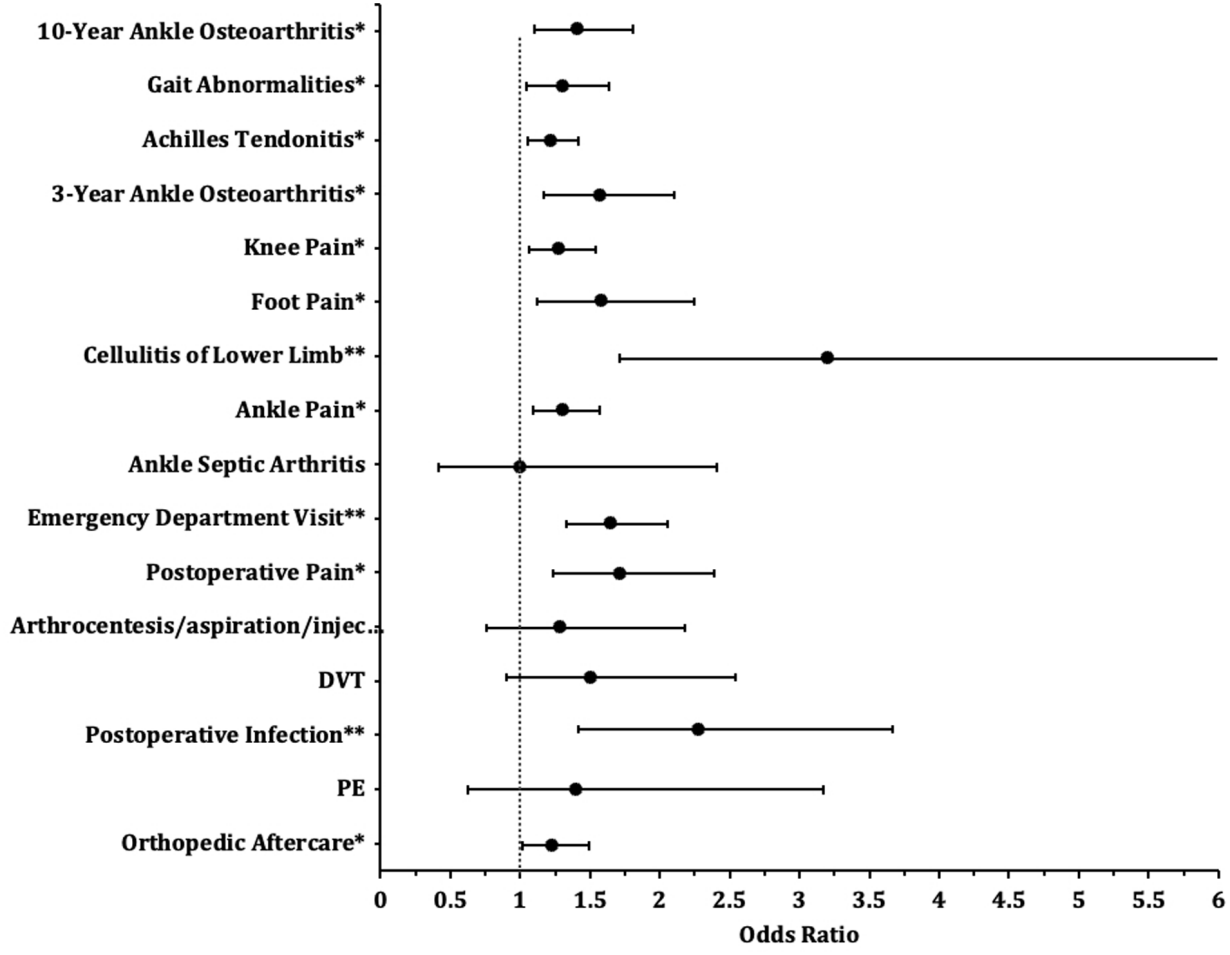
Odds ratios of postoperative outcomes by diagnosed mental health disorders. DVT, deep vein thrombosis; PE, pulmonary embolism.

Within 90 days after surgery, patients with mental health disorders demonstrated significantly higher odds of orthopaedic aftercare (OR 1.233, 95% CI 1.018-1.494, *P* = .032), infection following a procedure (OR 2.279, 95% CI 1.416-3.668, *P* < .0001), postoperative pain (OR 1.716, 95% CI 1.235-2.385, *P* = .001), emergency department visit (OR 1.653, 95% CI 1.328-2.058, *P* < .0001), ankle pain (OR 1.307, 95% CI 1.089-1.569, *P* = .004), cellulitis of the lower limb (OR 3.203, 95% CI 1.711-5.997, *P* < .0001), and foot pain (OR 1.582, 95% CI 1.117-2.241, *P* = .009). At 3 years postoperatively, patients with mental health disorders continued to show greater odds of knee pain (OR 1.282, 95% CI 1.065-1.543, *P* = .008), ankle osteoarthritis (OR 1.569, 95% CI 1.168-2.107, *P* = .003), Achilles tendonitis (OR 1.221, 95% CI 1.054-1.415, *P* = .008), and gait abnormalities (OR 1.305, 95% CI 1.043-1.633, *P* = .020). At 10 years, patients with mental health disorders remained at higher risk for developing ankle osteoarthritis (OR 1.412, 95% CI 1.102-1.808, *P* = .006) (Figure 1, Table 1). With the numbers available, no statistically significant difference was detected for pulmonary embolism, deep vein thrombosis, arthrocentesis/aspiration/injection, ankle septic arthritis, ACL rupture, repeat Achilles tendon repair, secondary Achilles tendon repair, short Achilles tendon, or calcific tendonitis of the Achilles.

**Table 1.** Outcomes Following Achilles Repair After 1:1 Propensity Matching of Patients With Diagnosed Mental Health Disorders (Mental Health) vs Patients Without Diagnosed Mental Health Disorders (Control).

Outcome	Mental Health (n = 1841)	Control (n = 1841)	Odds Ratio (95% CI)	*P* Value
90-d outcomes				
Orthopaedic aftercare	264	220	1.233 (1.018-1.494)	.032*
PE	14	10	1.403 (0.622-3.167)	.0413
Infection following a procedure	56	25	2.279 (1.416-3.668)	<.0001**
DVT	36	24	1.510 (0.897-2.541)	.118
Arthrocentesis/aspiration/injection	32	25	1.285 (0.758-2.177)	.350
Postoperative pain	99	59	1.716 (1.235-2.385)	.001*
ED visit	228	145	1.653 (1.328, 2.058)	<.0001**
Ankle septic arthritis	10	10	1 (0.415-2.408)	1
Ankle pain	304	242	1.307 (1.089-1.569)	.004*
Cellulitis of lower limb	41	13	3.203 (1.711-5.997)	<.0001**
Foot pain	84	54	1.582 (1.117-2.241)	.009*
3-y outcomes				
Knee pain	292	236	1.282 (1.065-1.543)	.008*
Ankle osteoarthritis	118	77	1.569 (1.168-2.107)	.003*
Achilles tendonitis	518	447	1.221 (1.054-1.415)	.008*
Gait abnormalities	191	150	1.305 (1.043-1.633)	.020*
ACL rupture	10	10	1 (0.415-2.408)	1
Repeat Achilles tendon repair	44	51	0.859 (.571-1.293)	.467
Secondary Achilles tendon repair	55	50	1.103 (0.748-1.627)	.621
10-y outcomes				
Ankle osteoarthritis	161	117	1.412 (1.102-1.808)	.006*
Short Achilles tendon	40	33	1.217 (0.764-1.938)	.408
Calcific tendonitis of the Achilles	16	16	1 (0.499-2.006)	1

Abbreviations: ACL, anterior cruciate ligament; DVT, deep vein thrombosis; ED, emergency department; PE, pulmonary embolism.

Categorical data are presented as n (% of cohort). Boldface indicates significance (*P* < .05).

** *P* < .0001; *P < .05.

## Discussion

The most important finding of this study is that preoperative mental health disorders (MHDs) were associated with significantly higher odds of both short- and long-term complications following Achilles tendon repair (ATR). Patients with depression or anxiety exhibited greater risks for early postoperative complications including infection, cellulitis, persistent pain, and increased health care utilization, as well as delayed associated diagnoses such as ankle osteoarthritis and gait abnormalities years after surgery. Given the retrospective design of this study, these findings should be interpreted as associative in nature, and future prospective investigations are warranted to better elucidate potential causal relationships. Importantly, several evaluated outcomes, including knee pain, ACL rupture, and gait abnormalities, are not Achilles-specific and should be interpreted as associated postoperative diagnoses rather than direct complications of Achilles tendon rupture or repair.

These results align with extensive literature linking psychological distress to poorer orthopaedic outcomes. Prior studies in trauma and arthroplasty populations consistently demonstrate that depression and anxiety predict worse functional recovery, higher pain scores, and increased complication rates.^11-13^ For example, large cohorts of total knee arthroplasty patients with preoperative depressive symptoms have shown greater infection risk, higher 30-day readmission rates, and poorer 1-year function compared with nondepressed counterparts.^12,13^ Likewise, in orthopaedic trauma settings, anxiety and depression independently predict slower rehabilitation and greater health care use.^11^ Our findings mirror these trends, extending the evidence to patients undergoing Achilles tendon repair, an area previously lacking large-scale data.

Within our cohort, patients with diagnosed MHDs demonstrated notably higher rates of infection, postoperative pain, and emergency department visits, compared with matched controls. These results reinforce that psychological health influences early surgical recovery, likely through both physiological and behavioral mechanisms such as altered stress responses and reduced adherence to rehabilitation protocols; however, further research is needed to clarify the underlying mechanisms driving these associations. In geriatric hip fracture populations, depression has similarly been linked to higher infection rates and prolonged hospitalization, paralleling our observation that MHDs complicate the immediate postoperative course in ATR patients.^14^

Importantly, this study also revealed significantly higher odds of long-term structural and functional diagnoses—including ankle osteoarthritis, Achilles tendinopathy, and gait abnormalities—among patients with MHDs at both 3 and 10 years postoperatively. Although prior work in orthopaedics often emphasizes patient-reported pain and satisfaction, our analysis underscores that mental health may also influence objective endpoints, such as joint degeneration and biomechanical dysfunction. This complements findings from arthroplasty research, where depressive or psychotic disorders have been associated with higher rates of revision surgery and long-term joint failure.^15^ Psychosocial stress may therefore contribute not only to subjective distress but also to tissue-level and biomechanical sequelae that manifest years later.^16,17^ There is no established mechanism by which Achilles tendon rupture or repair directly causes tibiotalar arthritis. Therefore, the observed association with ankle osteoarthritis should be interpreted cautiously and should not be considered evidence of causation.

Several interrelated mechanisms may explain the association between MHDs and poorer outcomes after ATR. First, altered pain processing and central sensitization are well documented in depression and anxiety.^18^ Heightened nociceptive signaling and impaired descending inhibition amplify pain perception, leading to greater postoperative pain and slower resolution.^19^ This could contribute to the increased rates of chronic ankle and foot pain observed in our MHD cohort. Second, neuroendocrine-immune dysregulation may impair tissue healing.^20,21^ Chronic stress elevates cortisol and proinflammatory cytokines, which can delay wound closure and increase infection susceptibility.^20^ Prior studies have shown that depressed patients exhibit impaired wound healing kinetics and prolonged inflammatory responses.^22^ These biologic mechanisms plausibly underlie the greater infection and cellulitis rates we identified. Third, behavioral and rehabilitative factors likely play a substantial role. Depression and anxiety often reduce motivation, energy, and adherence to postoperative protocols.^23^ Limited engagement in physical therapy, fear of reinjury, and kinesiophobia may hinder tendon remodeling and functional recovery, predisposing to gait abnormalities and eventual degenerative changes.^24^ The higher frequency of postoperative or emergency visits among MHD patients may reflect both genuine complications and increased symptom vigilance due to anxiety. Collectively, these physiological and behavioral pathways highlight how mental health influences surgical outcomes through multiple domains.

Clinically, these findings have several implications for perioperative management. First, routine mental health screening should be integrated into preoperative assessment for Achilles tendon repair. Brief validated tools such as the *PHQ-9* for depression and *GAD-7* for anxiety can identify at-risk patients who might otherwise go unrecognized.^25^ Second, preoperative counseling and expectation setting are essential. Patients with MHDs should be informed of their elevated risk for pain, delayed recovery, and potential long-term complications, helping them approach rehabilitation with realistic expectations and structured coping strategies.^9^ Third, multidisciplinary optimization may improve outcomes. Collaboration between orthopaedic surgeons, primary care providers, and mental health professionals can ensure that psychiatric conditions are actively managed before and after surgery.^26^ Evidence from arthroplasty cohorts suggests that cognitive-behavioral therapy and psychological support can reduce pain progression and improve postoperative function.^27,28^ Such interventions may be equally valuable in tendon repair populations.

Overall, these findings emphasize the importance of addressing psychological health as part of comprehensive surgical care. Given the number of outcomes assessed across multiple time windows, statistically significant findings should be interpreted cautiously, as no formal multiplicity correction was applied. Future prospective studies are needed to clarify the mechanisms underlying these associations and to determine whether targeted mental health interventions can improve both short- and long-term outcomes after Achilles tendon repair.

### Limitations

Although this study provides meaningful insight on the association between diagnosed mental health disorders and postoperative outcomes after Achilles tendon repair, it still possesses several limitations. Data obtained from large multicenter databases like TriNetX rely on the accuracy of diagnostic and procedural coding by participating health care organizations, which introduces the misclassification potential or incomplete data. Additionally, data sets from large databases are subject to temporal ambiguity and variability in documentation practices across institutions, which may influence the precision of diagnostic coding and outcome capture. Because TriNetX captures passive health care encounters rather than scheduled follow-up assessments, it was not possible to determine what proportion of outcomes occurred specifically near the 10-year time point or to confirm continuous observation for all patients through 10 years. This limitation may affect the reliability of long-term findings. Unmeasured confounding variables such as socioeconomic factors, medication adherence, and baseline functional status may still have influenced outcomes despite propensity score matching. Of the 15 905 eligible control patients, 1841 were retained after 1:1 propensity score matching, representing an exclusion of approximately 88% of the original control pool. This substantial reduction may introduce selection bias by limiting the matched cohort to a subgroup of controls whose characteristics were most similar to the mental health cohort, which may affect the generalizability of findings to the broader Achilles repair population without psychiatric comorbidity. Some patients with undiagnosed mental health conditions may have been included in the control cohort, which could bias the results. Moreover, our study did not account for operative parameters such as surgical approach, fixation technique, concurrent procedures, experience, or postoperative rehabilitation protocols. Patient-reported outcome measures were not available within the TriNetX database, limiting our ability to assess subjective pain, function, satisfaction, or perceived recovery following Achilles tendon repair. The mental health cohort also included a heterogeneous group of diagnoses, including depressive, anxiety, bipolar, and adjustment disorders, which may limit interpretation of diagnosis-specific effects. No power calculation was performed; therefore, the study may not have been adequately sized to detect or rule out differences in less frequent endpoints. Furthermore, the ability to establish a causal relationship between preexisting mental health diagnoses and postoperative complications is limited by the retrospective methodology. The generalizability of these findings may be impacted by the less well-defined underlying composition and documentation of the TriNetX database when compared with more established databases like the Nationwide Inpatient Sample (NIS) and the National Surgical Quality Improvement Program (NSQIP). Finally, diagnosed mental health status was identified using *ICD-10* codes rather than validated psychological assessment tools, which may not accurately reflect severity or treatment response. Although laterality can be specified for certain *CPT* and diagnosis codes, laterality capture is inconsistent across all postoperative diagnoses within the TriNetX platform, and restricting analyses to ipsilateral outcomes substantially reduced statistical power. As such, postoperative musculoskeletal diagnoses are best interpreted as patient-level associations observed over time rather than confirmed operative-side complications. As with all retrospective database analysis, these findings should be viewed as hypothesis generating and supportive of future prospective investigation rather than definitive evidence of causation. Because TriNetX is a federated electronic health record network rather than a closed longitudinal registry, follow-up reflects patient activity within participating institutions and may not capture encounters occurring outside the network. Despite these limitations, the results of this study underscore the influence mental health disorders may have on surgical recovery and emphasize the importance of incorporating psychosocial elements into perioperative planning and postoperative rehabilitation.

## Conclusion

Diagnosed mental health disorders are associated with increased short- and long-term adverse postoperative outcomes in patients who underwent Achilles repair at a population level. These associations included increased infection rates and pain, as well as later musculoskeletal diagnoses such as ankle osteoarthritis and abnormalities with gait.

## Supplemental Material

sj-pdf-1-fao-10.1177_24730114261457093 – Supplemental material for Impact of Diagnosed Mental Health Disorders on Postoperative Outcomes After Achilles Tendon Repair Supplemental material, sj-pdf-1-fao-10.1177_24730114261457093 for Impact of Diagnosed Mental Health Disorders on Postoperative Outcomes After Achilles Tendon Repair by Ahad A. Kesaria, Saadi M. Irfan, Tristan X. Nguyen, Jeffrey L. Shi, Verdinand C.B. Ruelos and William M. Weiss in Foot & Ankle Orthopaedics

## Appendix

**Supplementary Table 1.** Demographics of Patients With Diagnosed Mental Health Disorders (Cohort A) vs. Patients Without Diagnosed Mental Health Disorders (Cohort B) Before and After 1:1 Propensity Matching.

Before PSM						
Cohort	Demographic	Mean ± SD	Patients, n	% of Cohort	*P*	SMD
A	Age at index	49.1 ± 13.9	2114	100	<.001	0.438
B		42.8 ± 15.0	15 331	100		
A	Male		859	40.6	<.001	0.613
B			10 698	69.8		
A	Female		1065	50.4	<.001	0.522
B			3961	25.8		
A	White		1482	70.1	<.001	0.247
B			8946	58.4		
A	African American		241	11.4	<.001	0.227
B			3001	19.6		
A	Asian		30	1.4	<.001	0.149
B			581	3.8		
A	American Indian		10	0.5	.115	0.033
B			42	0.3		
A	Pacific Islander		10	0.5	.481	0.016
B			57	0.4		
A	Other race		71	3.4	.126	0.037
B			621	4.1		
A	Unknown race		274	13	.430	0.018
B			2083	13.6		
A	Not Hispanic or Latino		1553	73.5	.062	0.044
B			10 964	71.5		
A	Hispanic or Latino		119	5.6	.003	0.072
B			1137	7.4		
A	Unknown ethnicity		442	20.9	.866	0.004
B			3230	21.1		
A	Essential (primary) hypertension		916	43.3	<.001	0.640
B			2383	15.5		
A	Nicotine dependence		357	16.9	<.001	0.390
B			758	4.9		
A	Diabetes mellitus		385	18.2	<.001	0.412
B			801	5.2		
A	Obesity		771	36.5	<.001	0.642
B			1615	10.5		
A	Alcohol-related disorders		128	6.1	<.001	0.257
B			194	1.3		
A	Opioid-related disorders		45	2.1	<.001	0.176
B			36	0.2		
A	Osteoarthritis		731	34.6	<.001	0.534
B			1940	12.7		
A	Antidepressant use		1280	60.5	<.001	1.307
B			1306	8.5		
After PSM						
Cohort	Demographic	Mean ± SD	Patients, n	% of Cohort	*P*	SMD
A	Age at index	48.5 ± 14.0	1841	100	.025	0.074
B		49.6 ± 14.3	1841	100		
A	Male		819	44.5	.550	0.020
B			801	43.5		
A	Female		873	47.4	.869	0.005
B			868	47.1		
A	White		1291	70.1	.405	0.027
B			1314	71.4		
A	African American		218	11.8	.230	0.040
B			195	10.6		
A	Asian		29	1.6	.402	0.028
B			23	1.2		
A	American Indian		10	0.5	1	<0.001
B			10	0.5		
A	Pacific Islander		10	0.5	1	<0.001
B			10	0.5		
A	Other race		65	3.5	.717	0.012
B			61	3.3		
A	Unknown race		227	12.3	.619	0.016
B			237	12.9		
A	Not Hispanic or Latino		1358	73.8	.707	0.012
B			1368	74.3		
A	Hispanic or Latino		105	5.7	.613	0.017
B			98	5.3		
A	Unknown ethnicity		378	20.5	.902	0.004
B			375	20.4		
A	Essential (primary) hypertension		702	38.1	1	<0.001
B			702	38.1		
A	Nicotine dependence		251	13.6	.594	0.018
B			240	13		
A	Diabetes mellitus		278	15.1	.643	0.015
B			268	14.6		
A	Obesity		563	30.6	.518	0.021
B			545	29.6		
A	Alcohol-related disorders		80	4.3	.358	0.030
B			69	3.7		
A	Opioid-related disorders		21	1.1	.629	0.016
B			18	1		
A	Osteoarthritis		572	31.1	.410	0.027
B			549	29.8		
A	Antidepressant use		1007	54.7	.974	0.001
B			1006	54.6		

Abbreviations: PSM, propensity score matching; SMD, standardized mean difference.

**Supplementary Table 2.** Medical Codes for Included Diagnoses, Procedures, Events, and Medications.

Variable	Code
Mental health disorders	F32, F32.A, F33, F33.1, F34.1, F32.8, F33.2, F32.1, F33.0, F32.0, F32.2, F33.41, F31.30, F33.3, F32.4, F31.4, F31.32, F31.3, F43.21, F43.23, F33.9, F32.89, F32.9, F31.75, F32.3, F33.8, F31.31, F31.5, F31, F41.8, F40-48, F41.9, F41.1, F43.22, F40.8, F40.9
Any emergency department visit	*CPT*: 99284, *CPT*: 99283, *CPT*: 99285, *CPT*: 99282, *CPT*: 99281, *CPT*: 1013723, *CPT*: 1013711, HL7V3.0: VisitType: EMER, HCPCS: G0384, HCPCS: G0380, HCPCS: G0383, HCPCS: G0381, HCPCS: G0382
DVT	*ICD-10*: I82.4
PE	*ICD-10*: I26
Infection following a procedure	*ICD-10*: T81.4
Orthopaedic aftercare	*ICD-10*: Z47
Postoperative pain	*ICD-10*: G89.18
Ankle pain	*ICD-10*: M25.57
Cellulitis of lower limb	*ICD-10*: L03.119, L03.116, L03.115
Foot pain	*ICD-10*: M25.579
Arthrocentesis/aspiration/injection	*CPT*: 20610
Ankle septic arthritis	*ICD-10*: M00.87, M00.07
Knee pain	*ICD-10*: M25.56
Ankle osteoarthritis	*ICD-10*: M19.07
Achilles tendonitis	*ICD-10*: M76.6
Gait abnormalities	*ICD-10*: R26.81, R26
ACL Rupture	*ICD-10*: S83.509A
Repeat Achilles tendon repair	*CPT*: 27650
Secondary Achilles tendon repair	*CPT*: 27654
Short Achilles tendon	*ICD-10*: M67.0
Calcific tendonitis of the Achilles	*ICD-10*: M65.20

Abbreviations; ACL, anterior cruciate ligament; *CPT*, *Current Procedural Terminology*; DVT, deep vein thrombosis; ED, emergency department; *ICD-10*, *International Classification of Diseases, Tenth Revision*; PE, pulmonary embolism.
